# Nitrous Oxide-Induced Pancytopenia and Peripheral Neuropathy From Chronic Whip-It™ Use: A Case Report

**DOI:** 10.7759/cureus.112883

**Published:** 2026-07-17

**Authors:** Shannon Pierce, Sarah Gergis, Sophia Dragan, Sai Sushrutha Mudupula Vemula, Borys Hrinczenko

**Affiliations:** 1 Internal Medicine, McLaren Greater Lansing, Lansing, USA; 2 Hematology and Medical Oncology, McLaren Greater Lansing, Lansing, USA; 3 College of Medicine, Michigan State University, East Lansing, USA; 4 Internal Medicine, University of Michigan Health Sparrow Hospital, Lansing, USA

**Keywords:** anemia, cartridge, megaloblastic, myeloneuropathy, neuropathy, nitric, oxide, pancytopenia, rapid, whip-it

## Abstract

Nitrous oxide (N_2_O) is a colorless, odorless gas gaining popularity among recreational drug users for its euphoric effects. Whip-it!™ is a low-cost, freely available form of N_2_O commonly abused in parties and nightclubs. Chronic N_2_O use can inactivate vitamin B12, causing severe deficiency, anemia, neuropathy, and myelopathy. Here, we report a 19-year-old female with no significant past medical history who was found unresponsive in her apartment. She admitted to daily Whip-it™ use over the preceding three months. In the emergency department, she reported generalized weakness and tingling in all extremities. Workup revealed pancytopenia and profound vitamin B12 deficiency. She was treated with vitamin B12 supplementation and blood transfusions, with significant symptom improvement by discharge. N_2_O inactivates vitamin B12 through irreversible oxidation of the cobalt atom, causing deficiency and neurologic injury. Unlike other classic causes of vitamin deficiency, such as a vegan diet or pernicious anemia, N_2_O-induced anemia can occur very rapidly (within days or weeks). Clinicians should consider recreational N_2_O use in young patients presenting with cytopenias and neurologic symptoms. Given its easy availability and rising misuse, public health education to raise awareness is needed.

## Introduction

Nitrous oxide (N_2_O), colloquially known as "laughing gas," has a long history of medical use, particularly in anesthesia and analgesia during dental procedures and labor. However, beyond its clinical applications, N_2_O has emerged as a popular recreational drug due to its dissociative and euphoric properties. Recreational users often inhale N_2_O from small, pressurized cartridges called whippets or Whip-its™, originally intended for whipping cream dispensers. One cartridge can contain up to four liters of gas. These cartridges are legally sold in stores and online, often with minimal regulation and without age restrictions [1].

In a report by Jeffries, the gas's widespread availability and social media presence have significantly contributed to its normalization in youth culture [2]. This normalization has led to underestimation of the associated health risks, which include irreversible neurologic damage and hematologic complications.

## Case presentation

A 19-year-old female with no known past medical history presented to the emergency room via ambulance after being found unresponsive, covered in emesis, and with Whip-it™ canisters nearby. In the emergency department (ED), she was lethargic but arousable and oriented. She reported generalized weakness and tingling in her extremities and admitted to using Whip-its daily for the past three months.

Vital signs were stable. Examination revealed dry mucosa, poor dentition, and inflamed nasal turbinates. On neurological exam, she had reduced 4/5 strength in the bilateral upper/distal lower extremities, reduced 3/5 strength in the bilateral proximal lower extremities, and she reported decreased sensation in her fingers and toes to light touch. She also had a flat affect. Proprioception and vibration testing were not assessed upon evaluation in the ED and admission to the hospital due to the patient's lethargy and lack of appropriate tools for testing at presentation, but would have been helpful for ascertaining involvement of the patient's posterior/dorsal column.

Lab results are noted in Table 1. Patient's labs were ultimately remarkable for pancytopenia, normal mean corpuscular volume (MCV), normal iron studies, low B12 level with elevated methylmalonic acid level, and markers of hemolysis (elevated lactate dehydrogenase (LDH) and low haptoglobin). The urine drug screen was negative.

**Table 1:** Patient lab results Labs are notable for pancytopenia with normal MCV and elevated methylmalonic acid level. The patient also had normal iron studies and markers suggestive of hemolysis, such as low haptoglobin and elevated LDH.

Lab Test	Value	Reference Range
White blood cell (WBC) count	1.5 x 10^3^/µL	4.5-10 x 10^3^/µL
Absolute neutrophil count (ANC)	0.34 x 10^3^/µL	1.8-7.7 x 10^3^/µL
Hemoglobin (Hb)	6.2 g/dL	12-15 g/dL
Mean corpuscular volume (MCV)	82 fL	80-97 fL
Platelet count	51 x 10^3^/µL	140-440 x 10^3^/µL
Reticulocyte count	0 x 10^6^/µL	0.01-0.08 x 10^6^/µL
Haptoglobin	<15 mg/dL	31.2-198 mg/dL
Lactate dehydrogenase (LDH)	1591 U/L	120-246 U/L
Folate level	>24 ng/mL	4.4-31 ng/mL
Vitamin B12 level	104 pg/mL	200-944 pg/mL
Methylmalonic acid	0.44 nmol/mL	0.0-0.4 nmol/mL
Iron level	74 µg/dL	50-170 µg/dL
Ferritin	387 ng/mL	10-291 ng/mL

Peripheral smear revealed normocytic anemia with marked neutropenia and thrombocytopenia, without immature cells. CT and MRI of the brain were negative. The patient refused a cervical spine MRI.

Our patient was diagnosed with N_2_O-induced vitamin B12 deficiency, which led to pancytopenia and neurologic symptoms. She received 1,000 mcg of intramuscular (IM) vitamin B12 daily for one week, followed by weekly injections. Two units of packed red blood cells were transfused. Psychiatry was consulted, and she was provided with substance abuse resources. Upon discharge, her hemoglobin improved to 11.0 g/dL, absolute neutrophil count (ANC) to 0.65 x 10^3^/µL, and vitamin B12 to 1407 pg/mL.

## Discussion

**Figure 1:**
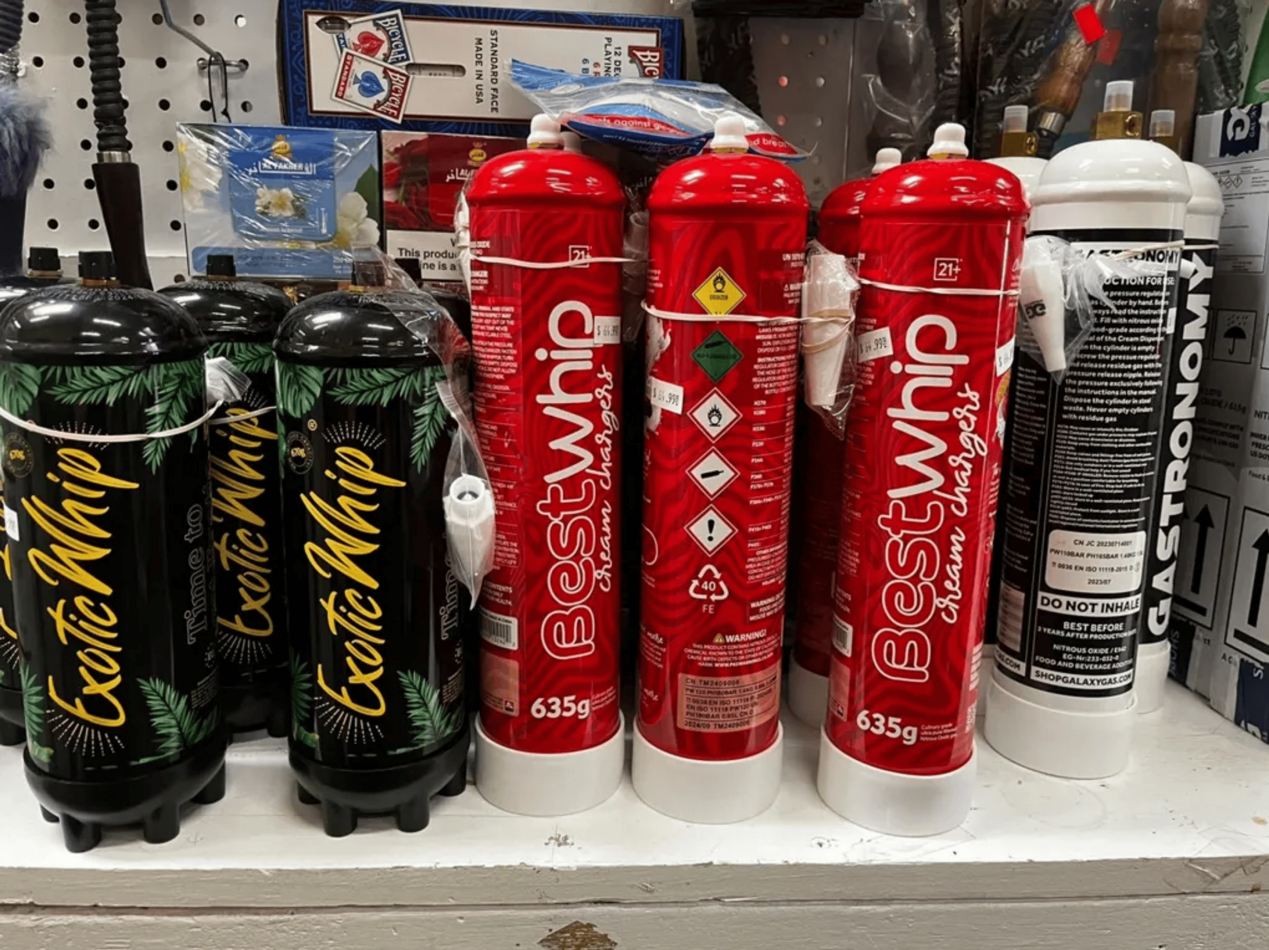
Commercial nitrous oxide (“Whip-it™”) canisters commonly sold in convenience stores and online. These large 635 g cartridges, originally intended for whipped cream dispensers, are easily accessible and often misused recreationally by young adults. Image Source: Reference [6]

N_2_O-induced neurologic injury was first reported in 1978, when it was associated with neuropathy following chronic abuse [3,4]. N_2_O, a colorless, sweet-tasting gas first synthesized in 1775, has long been used as an anesthetic agent, especially in dentistry and obstetrics [5]. However, its dissociative and euphoric properties have made it an increasingly popular recreational drug, particularly in the form of whipped cream canisters, tanks, or small cartridges called "whippets" (Figure 1) [6]. Easy access - both online and at nightclubs - has facilitated its widespread misuse [3,7].

In the case of our patient presenting with altered mental status and peripheral neuropathy in the setting of regular Whip-it™ use over three months, and lab findings of pancytopenia with low vitamin B12 levels, N_2_O-induced B12 deficiency was the most likely cause of her symptoms. Other differentials to consider would be other nutritional deficiencies, such as vitamin B1/B6 deficiency, copper deficiency, iron deficiency, or zinc deficiency. Additionally, neurologic syndromes, such as seizure, stroke, or multiple sclerosis, were also considered with respect to her peripheral neuropathy and altered mentation, but were considered less likely given unremarkable MRI results and improvement in symptoms after replenishment of vitamin B12 stores.

**Figure 2:**
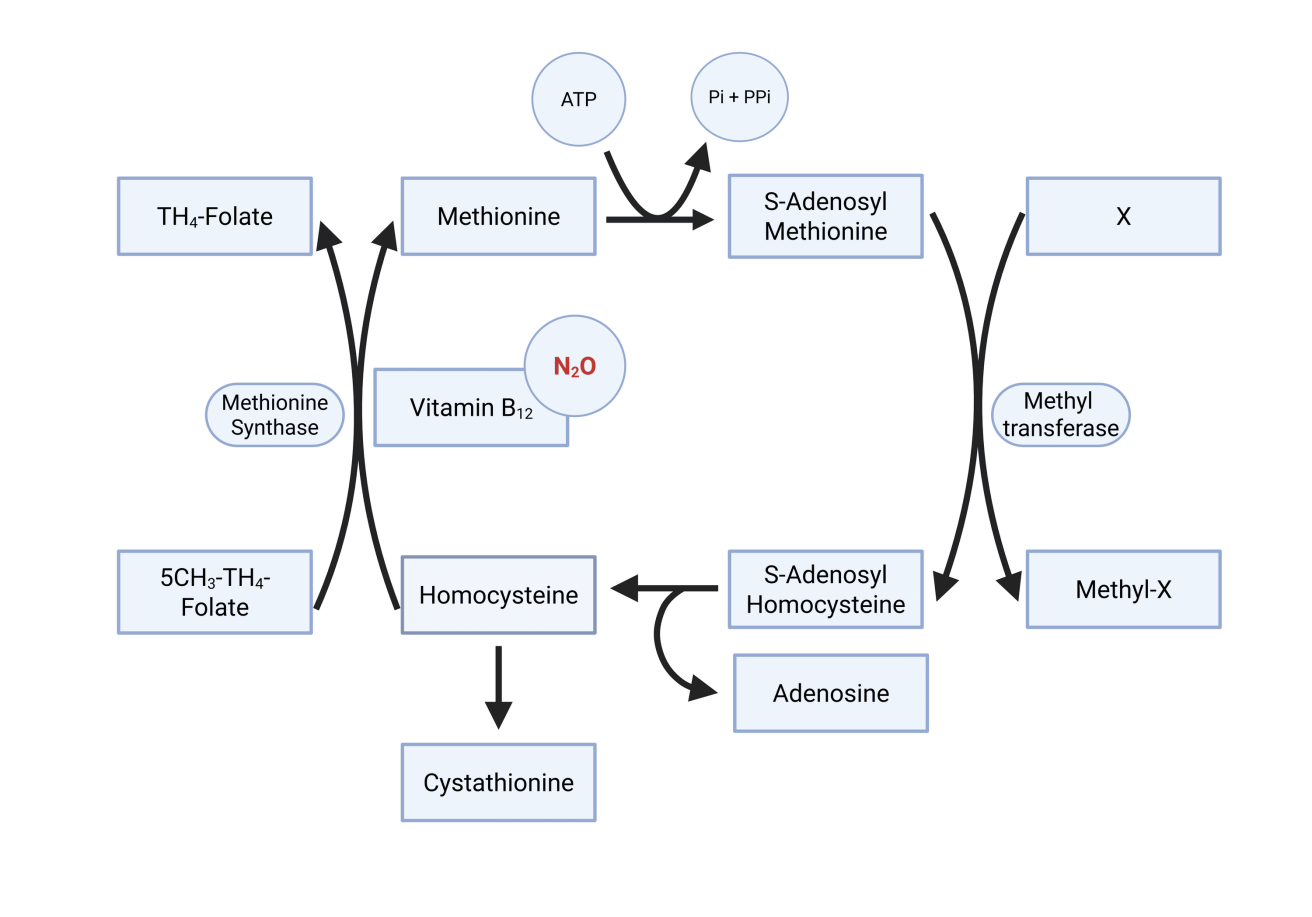
Nitrous oxide interference with vitamin B12 metabolism and irreversible inactivation of methionine synthase. The above figure details how N_2_O-induced B12 deficiency impairs conversion of homocysteine to methionine (as B12 is a necessary cofactor for methionine synthase). This ultimately reduces production of S-adenosylmethionine, which is required for methylation of myelin phospholipids to maintain their stability. Failure of adequate methylation of myelin phospholipids drives the pathophysiology of subacute combined degeneration of the spinal cord seen in B12 deficiency. Figure created by the authors using BioRender (Science Suite Inc., Toronto, ON, Canada).

The pathophysiology of N_2_O-induced neurologic injury involves interference with vitamin B12 metabolism. Vitamin B12 is essential for the formation of methionine via the methylation of homocysteine (Figure 2). In turn, methionine is converted to S-adenosylmethionine (SAM), which is required for all methylation reactions, including those of myelin phospholipids (Figure 2) [8,9]. When methylation of myelin fails to occur, demyelination within the central and peripheral nervous system occurs, which explains the neurologic symptoms associated with B12 deficiency [9]. N_2_O interacts with vitamin B12 by irreversibly oxidizing the cobalt ion of the cobalamin on vitamin B12, resulting in the deactivation of vitamin B12 [10]. Oxidation of the cobalt ion by N_2_O prevents methylcobalamin from acting as a coenzyme in methionine production and subsequently SAM production [11].

In contrast to other causes of vitamin B12 deficiency (such as pernicious anemia or dietary insufficiency), N_2_O-induced B12 deficiency can develop rapidly, within days to weeks of repeated exposure [7,12,13]. Classically, it is associated with the lab finding of macrocytic anemia, although this may not always be present, as is the case for our patient. Other symptoms of vitamin B12 deficiency include neurocognitive changes, glossitis or (less commonly) skin hyperpigmentation, and osteoporosis [3,7,14]. Another possible complication of B12 deficiency includes pancytopenia, as seen in our patient, because low levels of B12 cause decreased methylation of DNA and synthesis of nucleotides, which can decrease production of all blood cell lines [15].

As a result of the interaction between N_2_O and vitamin B12, vitamin B12 levels can drop rapidly, resulting in the quick development of symptoms of subacute combined degeneration (SCD) of the spinal cord. This is how N_2_O-induced B12 deficiency differs from other etiologies of vitamin B12 deficiency (pernicious anemia or vegan diet), which can take many years before symptoms develop. Vitamin B12 is an important cofactor for two enzymes involved in the preservation of myelin, methionine synthase and methylmalonyl-CoA mutase [16]. SCD is primarily driven by demyelination of the dorsal columns and is characterized by dysesthesia or neuropathic pain, gait ataxia, impaired position/vibration sense, and spastic weakness in the lower extremity or all four extremities. In a number of cases, individuals have also presented with urinary retention [16]. MRI studies will generally show hyperintense foci within the dorsal columns on T2-weighted imaging, although there are some cases reported where MRI did not detect any abnormalities of the spine despite the presence of neurologic symptoms in the patients [14,17]. In the case of our patient, it is unclear whether her N_2_O-induced B12 deficiency caused SCD of the spinal cord, given the lack of key neurological physical exam testing, such as proprioception/vibration testing, and the personal choice to decline spinal MRI.

Treatment of vitamin B12 deficiency and N_2_O toxicity is vitamin B12 replenishment. Generally, this can be given via IM or oral formulations. Intravenous cyanocobalamin is not used. For patients with neuropsychiatric symptoms, malabsorption concerns, or severe anemia, IM cyanocobalamin is preferred as initial therapy in practice, usually given as 1000 mcg daily for one to two weeks, then weekly for four weeks, then monthly thereafter. Research suggests that for most cases of B12 deficiency, daily oral B12 is similarly efficacious at raising serum B12 levels when compared to IM cyanocobalamin and potentially more effective than IM formulations at doses of 1000-2000 mcg per day [18]. Treatment for SCD should be aggressive/rapid, and although laboratory values improve quickly, it may take 3-12 months for the neurological deficits to improve [16]. While some case reports have described the reversibility of neurological deficits with the cessation of N_2_O use and vitamin B12 repletion as in our case [13], others have found the neurological deficits to be persistent, only partially reversible, or irreversible [7,17,19].

Studies show that vitamin B12 deficiency can cause divergent phenotypes, like isolated cytopenias, neurologic impairment, or both, depending on which metabolic pathway is affected. B12 is essential for methionine synthase in the cytosol, which is required for DNA synthesis, especially in rapidly proliferating hematopoietic cells. Another key enzyme that requires vitamin B12 as a coenzyme, methylmalonyl-CoA mutase, is located in the mitochondria and is essential for fatty acid metabolism and myelin stability [20]. Deficiency can thus impair red cell production (megaloblastic anemia) or neuronal integrity (neuropathy), which is also influenced by folate status, duration of deficiency, and individual variation in enzyme sensitivity and tissue demand [21]. Neurologic symptoms often occur when folate is adequate, as impaired DNA synthesis drives hematologic changes while methylmalonic acid accumulation and altered methylation cause neurologic damage, and folate supplementation masks hematologic features while worsening neurologic symptoms [22]. Early or acute deficiencies may first appear hematologic, while chronic or insidious depletion often leads to isolated neurologic disease [21-23]. This clinical divergence was first systematically documented by Lindenbaum et al. (1988), who found that 28% of B12-deficient patients with neurologic disease lacked anemia or macrocytosis, showing that hematologic and neurologic pathways can be independently affected [24].

Additionally, the findings of hemolysis seen in our patient have been documented in other case reports. The prevailing thought is that hemolytic anemia associated with N_2_O use may be a consequence of N_2_O-induced oxidative stress, which ultimately causes lysis of the red blood cells. Also, rapid depletion of B12 stores from N_2_O binding may result in intramedullary hemolysis of immature red blood cells in the marrow [25].

Beyond the clinical implications, N_2_O abuse carries significant societal consequences given the accessibility and appeal of Whip-its™ to young teens/adults in the current time. Given the increasing recreational use, particularly among vulnerable populations, healthcare providers must remain vigilant and keep N_2_O use/Whip-it™ use on the differential in patients presenting with signs/symptoms of B12 deficiency. Public health officials should raise awareness of these potential side effects. Early recognition and counseling can significantly reduce the morbidity associated with N_2_O toxicity.

## Conclusions

This case highlights N_2_O-induced vitamin B12 deficiency as an important diagnostic consideration in young patients presenting with neurologic symptoms and hematologic abnormalities, including pancytopenia. Recreational Whip-it™ use can cause rapid functional vitamin B12 deficiency, and early recognition is essential to prevent potentially irreversible neurologic injury. Prompt cessation of N_2_O exposure, vitamin B12 repletion, supportive care, and counseling regarding substance use can lead to meaningful clinical and hematologic improvement.
